# Evaluation of tolvaptan-associated hepatic disorder using different national pharmacovigilance databases

**DOI:** 10.1038/s41598-024-77052-y

**Published:** 2024-10-29

**Authors:** Takaya Uno, Kouichi Hosomi, Satoshi Yokoyama

**Affiliations:** Division of Drug Informatics, School of Pharmacy, Kindai University, 3-4-1 Kowakae, Higashi-Osaka, Osaka, 577-8502 Japan

**Keywords:** Tolvaptan, Hepatic disorder, Liver, Spontaneous reporting system, Adverse event, Pharmacovigilance, Hepatitis, Hepatotoxicity, Liver cirrhosis, Liver fibrosis, Polycystic kidney disease, Heart failure, Hepatitis, Hepatotoxicity, Liver cirrhosis, Liver fibrosis, Epidemiology, Polycystic kidney disease, Risk factors, Digestive signs and symptoms

## Abstract

Tolvaptan-associated hepatic disorder is a rare, but lethal adverse event; however, the precise risk and time of onset remain unclear. This study aimed to characterize the severity, time‑to‑onset, and outcomes of hepatic disorder based on patient age and sex. Patient data were acquired from the Japanese Adverse Drug Event Report database (JADER) and the JAPIC AERS database, which consists of the U.S. Food and Drug Administration Adverse Event Reporting System (FAERS) processed by the Japan Pharmaceutical Information Center. Hepatic disorder was classified as severe or nonsevere. Tolvaptan use was associated with hepatic disorder in analyses using the FAERS [Severe hepatic disorder: reporting odds ratio (ROR) 4.93, 95% confidence interval (CI) 4.33‒5.61; information component (IC) 2.11, 95% CI 1.92‒2.29; nonsevere hepatic disorder: ROR 6.78, 95% CI 6.01‒7.65; IC 2.51, 95% CI 2.33‒2.68] and the JADER (severe hepatic disorder: ROR 4.21, 95% CI 3.57‒4.97; IC 1.86, 95% CI 1.63‒2.10; nonsevere hepatic disorder: ROR 4.27, 95% CI 3.68‒4.95; IC 1.83, 95% CI 1.62‒2.04). A time‑to‑onset analysis revealed that the median onset time was significantly longer in patients aged < 60 years compared with patients aged ≥ 60, regardless of the severity (FAERS: severe hepatic disorder 7 vs. 58 days, *p* < 0.0001; nonsevere hepatic disorder 8 vs. 52.5 days, *p* < 0.0001; JADER: severe hepatic disorder 9.5 vs. 32 days, *p* = 0.0017; nonsevere hepatic disorder 9 vs. 89 days, *p* < 0.0001). Severe outcomes were observed, regardless of the severity of hepatic disorder. Patients should be monitored for liver function based on age to prevent fatal outcomes.

## Introduction

Tolvaptan, a vasopressin receptor 2 antagonist, is widely used to treat autosomal dominant polycystic kidney disease, syndrome of inappropriate secretion of antidiuretic hormone, and edema caused by heart failure and cirrhosis^1,2^. However, it is necessary to appropriately manage adverse events (AEs), which include thirst, hypernatremia, constipation, and polyuria^3–6^. Thirst is the most frequently reported AE, and adequate hydration is necessary to avoid dehydration. In particular, elderly patients become less sensitive to thirst are require more attention^7^. Recently, the results of a survey using the Japanese Adverse Drug Event Report database (JADER), indicated that tolvaptan had a high association with drug‐induced liver injury^8^. Hepatic disorder caused by tolvaptan is a rare, but potentially lethal AE^9^. There are some reports indicating that tolvaptan is associated with idiosyncratic drug-induced liver injury^9–15^, and regular hepatic monitoring is recommended to prevent liver damage^10^. The mechanisms underlying tolvaptan-associated hepatoxicity have been examined in vitro^16–20^, and hypersensitivity to tolvaptan has been associated with liver injury^17^. In the clinical study, the association between female sex and hepatoxicity has been reported, but this report has not been thoroughly investigated due to the small number of cases^15^. Furthermore, the impact of aging on tolvaptan-associated hepatoxicity has not been thoroughly studied. In a previous research on various drugs excluding tolvaptan, aging and female sex have been identified as risk factors for drug‐induced liver injury^21^. Therefore, aging and sex may influence the onset of tolvaptan-associated hepatic disorder, though the relationship remains unclear.

Pharmacovigilance analysis is an important tool for the postmarketing surveillance of drug safety. Databases of spontaneous AE reports, such as the U.S. Food and Drug Administration (FDA) Adverse Event Reporting System (FAERS) provided by the FDA and the JADER provided by the Pharmaceuticals and Medical Devices Agency in Japan, contain large amounts of data and are used in pharmacovigilance analyses^22–26^. Pharmacovigilance analysis using FAERS and JADER is important to understand the occurrence of AEs in clinical practice without the limitations of clinical trials, such as enrollment criteria, small sample size, and limited follow-up period. Therefore, we examined the relationship between tolvaptan-associated hepatic disorder and aging or sex using the FAERS and JADER.

## Methods

### Data sources

We used two spontaneous AE reporting databases constructed by regulatory authorities in the United States of America and Japan. FAERS and JADER contain reports of spontaneous AEs reported by consumers, pharmaceutical companies, and medical institutions around the world and in Japan, respectively. We analyzed FAERS and JADER records between April 2004 and March 2022. We used data from JAPIC AERS, which was processed by the Japan Pharmaceutical Information Center (JAPIC) to analyze FAERS. FAERS consists of seven datasets: patient demographic and administrative information (DEMO), drug information (DRUG), AEs (REAC), indications for the use of reported drugs (INDI), start and end dates for the reported drugs (THER), outcomes for the event (OUTC) and sources for the event (RPSR). JADER consists of four datasets: patient demographic and administrative information (DEMO), drug information (DRUG), AEs (REAC), and primary disease (HIST). We removed duplicate cases using reported patient information. Reports with unclear or missing data with respect to age, sex, patient outcome, onset date of AE occurrence, and initial date of drug administration, were excluded from FAERS and JADER. Also, the study included patients aged ≥ 0, as tolvaptan is commonly used in children^27^. Finally, a total of 8,444,876 drug-reaction pairs were identified from 1,454,162 FAERS reports, while 818,050 drug-reaction pairs were identified from 349,900 JADER reports.

### Identification of the target drug

In FAERS, the contribution of drugs to AEs is classified as “Primary suspect drug,” “Secondary suspect drug,” “Concomitant,” and “Interacting.” Reports classified as “Primary suspect drug” or “Secondary suspect drug” were extracted. Reports in FAERS contain the name of the medical product and the active ingredient. JAPIC AERS contains JAPIC dictionary files, which include the substance name as the drug name, based on the name of the medical product and active ingredient. We extracted reports including “Tolvaptan” as the substance name. For JADER, the contribution of drugs to AEs is classified as “Suspect drug,” “Concomitant drug,” and “Interaction.” We restricted the drug role as “Suspect drug.” Because reports in JADER have trade names and generic names, we extracted reports including “Tolvaptan” as the generic name.

### Definition of AEs

AEs in FAERS and JADER are coded using preferred terms in the Medical Dictionary for Regulatory Activities (MedDRA) terminology. We used MedDRA version 26.0 for the analysis. The standardized MedDRA queries (SMQs) contain groups of preferred terms related to a defined medical condition or area of interest. We used “Drug related hepatic disorders–comprehensive search (SMQ: 20000006).” The layer below “Drug related hepatic disorders–comprehensive search” contained “Drug related hepatic disorders–severe events only (SMQ: 20000007),” “Liver related investigations, signs and symptoms (SMQ: 20000008),” “Cholestasis and jaundice of hepatic origin (SMQ: 20000009),” and “Liver-related coagulation and bleeding disturbances (SMQ: 20000015).” We defined “SMQ: 20000007” as “Severe hepatic disorder.” Moreover, “SMQ: 20000008,” “SMQ: 20000009,” and “SMQ: 20000015” were defined as “Nonsevere hepatic disorder.” Reports including the preferred terms “Severe hepatic disorder” and “Nonsevere hepatic disorder” were classified as reports including the preferred term “Severe hepatic disorder.”

### Trends in outcomes

A mosaic plot was constructed to visually evaluate the relationship between severe or nonsevere hepatic disorder and outcomes in patients treated with tolvaptan. In FAERS, the outcome that caused AEs were classified as “Death,” “Life-threatening,” “Hospitalization,” “Disability,” “Congenital anomaly,” “Required intervention to prevent permanent impairment/damage,” and “Other serious.” Reports with outcomes classified as “Congenital anomaly” and “Required intervention to prevent permanent impairment/damage” did not exist for patients treated with tolvaptan. In JADER, the outcome caused AEs were classified as “Death,” “Not recovered,” “With sequelae,” “Remission,” and “Recovered.” As patients reported ≥ 1 outcome, the total number of outcomes is not necessarily equal to the total number of patients.

### Statistical analyses

#### Disproportionality analysis

The rate of AEs cannot be calculated using FAERS and JADER because these databases are constructed based on spontaneous reports. Therefore, the association between tolvaptan and AEs was determined using a case/noncase analysis to examine whether suspected AEs (cases) are differentially reported with tolvaptan compared with other AEs (noncases)^28^. As disproportionate indicators, we used the reporting odds ratio (ROR) and information component (IC) to reduce the likelihood of false positive signals^29^. The ROR and IC are common methods used by the Netherlands Pharmacovigilance Center and the World Health Organization-Uppsala Monitoring Center, respectively^30–32^. AEs were considered drug-associated when the lower limit of the 95% confidence interval (CI) of ROR > 1 and the lower limit of the 95% CI of IC > 0^29^.

#### Multiple logistic regression analysis

Based on previous reports, age and sex were considered confounding factors for tolvaptan-induced AEs^7,15^. Because age is a continuous variable, we categorized patients as nonelderly patients < 60 years or elderly patients ≥ 60 years. When considering the confounding factors, we also performed a multiple logistic regression analysis. The objective variable was designated severe hepatic disorder or nonsevere hepatic disorder. The explanatory variable was age (< 60 years or ≥ 60 years) and sex (male or female) and these factors were forced entry.

#### Time‑to‑onset analysis

The period from the initial date of drug administration to the onset date of AEs was defined as the time-to-onset of AEs caused by the drug. Therefore, the onset time of AEs was calculated as “(Onset date of AEs) − (Initial date of drug administration).” Reports with onset time less than 0 were excluded. The Weibull shape parameter and the median days to onset were used to clarify the time-to-onset profile for AEs^33,34^. The Weibull distribution includes the scale parameter α and shape parameter β. For the scale parameter α, a larger scale value indicates a wider data distribution, whereas a smaller scale value indicates a narrower data distribution. The value of the shape parameter shows the distribution of failure rate over time, which is classified as follows: (1) early failure type with an upper limit of the 95% CI of the β value less than 1; (2) random-failure type with 95% CI of the β value including 1; and (3) wear-out-failure type with a lower limit of the 95% CI of the β value greater than 1. Cumulative incidences of severe or nonsevere hepatic disorder were plotted using the Kaplan–Meier method. A generalized Wilcoxon test was used to compare the cumulative incidences of severe or nonsevere hepatic disorder between patients aged < 60 years or ≥ 60 years treated with tolvaptan.

In this study, data management and statistical analyses were performed using Alkano (version 1.2.2; Mathematical Systems Inc., Tokyo, Japan) and JMP pro (version 17.0.0; SAS Institute Inc., Cary, NC, USA). A *p* value less than 0.05 was considered statistically significant.

## Results

### Characteristics of patients for severe or nonsevere hepatic disorder related to tolvaptan

The FAERS contained a total of 46,489 and 42,580 drug-reaction pairs for severe and nonsevere hepatic disorder, respectively. Additionally, the JADER had a total of 19,041 drug-reaction pairs for nonsevere hepatic disorder and 14,106 for severe disorder. In the FAERS and JADER, Table 1 displays the characteristics of patients with severe or nonsevere tolvaptan-related hepatic diseases. Patients aged < 20 years made up less than 1% of all cases in both FAERS and JADER, regardless of severity. The proportions of patients aged ≥ 60 years were greater except for individuals with nonsevere hepatic disorder in the FAERS (FAERS: severe hepatic disorder 59.6%, nonsevere hepatic disorder 38.9%; JADER: severe hepatic disorder 75.4%, nonsevere hepatic disorder 59.4%). The proportions of males were greater, regardless of severity (FAERS: severe hepatic disorder 55.5%, nonsevere hepatic disorder 55.1%; JADER: severe hepatic disorder 63.5%, nonsevere hepatic disorder 54.8%). Congenital cystic kidney disease accounted for the majority of all indications except for individuals with severe hepatic disorder in the JADER (FAERS: severe hepatic disorder 16.2%, nonsevere hepatic disorder 20.2%; JADER: nonsevere hepatic disorder 30.3%). Meanwhile, there were numerous reports with unclear indications (FAERS: severe hepatic disorder 36.2%, nonsevere hepatic disorder 55.3%; JADER: severe hepatic disorder 7.0%, nonsevere hepatic disorder 16.3%). Supplementary Tables S1 and S2 contain a list of all the indications for severe and nonsevere hepatic disorder associated with tolvaptan.

**Table 1:** Characteristics of patients for severe or nonsevere hepatic disorder related to tolvaptan.

FAERS	Value (%)	JADER	Value (%)
Severe hepatic disorder		Severe hepatic disorder	
Number of patients	265	Number of patients	167
Age (years)		Age (years)	
< 20	1 (0.4)	< 20	1 (0.6)
20–59	106 (40.0)	20–59	40 (24.0)
≥ 60	158 (59.6)	≥ 60	126 (75.4)
Sex		Sex	
Female	118 (44.5)	Female	61 (36.5)
Male	147 (55.5)	Male	106 (63.5)
Indications (top three)		Indications (top three)	
Congenital cystic kidney disease	43 (16.2)	Fluid retention	35 (20.5)
Fluid retention	39 (14.7)	Edema due to hepatic disease	25 (14.6)
Cardiac failure	17 (6.4)	Cardiac failure	20 (11.7)
Unknown	96 (36.2)	Unknown	12 (7.0)
Nonsevere hepatic disorder		Nonsevere hepatic disorder	
Number of patients	321	Number of patients	219
Age (years)		Age (years)	
< 20	3 (0.9)	< 20	2 (0.9)
20–59	193 (60.1)	20–59	87 (39.7)
≥ 60	125 (38.9)	≥ 60	130 (59.4)
Sex		Sex	
Female	144 (44.9)	Female	99 (45.2)
Male	177 (55.1)	Male	120 (54.8)
Indications (top three)		Indications (top three)	
Congenital cystic kidney disease	65 (20.2)	Congenital cystic kidney disease	67 (30.3)
Cardiac failure	20 (6.2)	Cardiac failure	38 (17.2)
Fluid retention	14 (4.3)	Cardiac failure congestive	18 (8.1)
Unknown	178 (55.3)	Unknown	36 (16.3)

Because patients reported two indications, the total number of indications does not always correspond to the total number of patients.

FAERS; FDA Adverse Event Reporting System, JADER; Japanese Adverse Drug Event Report database.

### Trends in outcomes

**Fig. 1:**
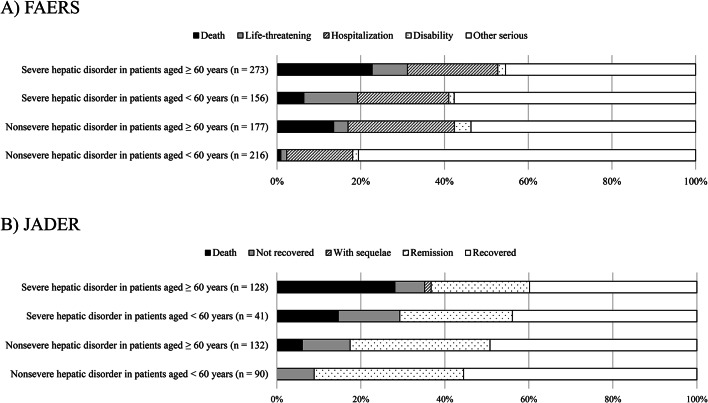
Mosaic plot of outcomes for severe or nonsevere hepatic disorder stratified by age in the FAERS and JADER. The total number of outcomes may not necessarily match the total number of patients because patients reported ≥ 1 outcome. Severe outcomes such as “death,” “life-threatening,” “hospitalization,” and “not recovered” were included in the FAERS and JADER.

We used mosaic plots to describe the outcome profiles for severe or nonsevere hepatic disorder stratified by age in FAERS and JADER (Fig. 1). In FAERS, the rates of “Death” outcomes was >20% for severe hepatic disorder in patients aged ≥ 60 years [22.7% (62/273 cases)], while the rate of “Death” outcomes was low for nonsevere hepatic disorder in patients aged < 60 years [6.4% (10/156 cases)]. Outcomes for nonsevere hepatic disorder in patients aged < 60 years included “Hospitalization” [15.7% (34/216 cases)]. In JADER, the rates of “Death” outcomes were > 20% for severe hepatic disorder in patients aged ≥ 60 years [28.1% (36/128 cases)], while the “Death” outcomes were not included for nonsevere hepatic disorder in patients aged < 60 years. Outcomes for nonsevere hepatic disorder in patients aged < 60 years, outcomes contained “Not recovered” [8.9% (8/90 cases)].

### Disproportionality analysis

Table 2 shows the results of a disproportionality analysis of tolvaptan and severe or nonsevere hepatic disorder in FAERS and JADER (FAERS: severe hepatic disorder; ROR 4.93, 95% CI 4.33‒5.61; IC 2.11, 95% CI 1.92‒2.29; nonsevere hepatic disorder; ROR 6.78, 95% CI 6.01‒7.65; IC 2.51, 95% CI 2.33‒2.68; JADER: severe hepatic disorder; ROR 4.21, 95% CI 3.57‒4.97; IC 1.86, 95% CI 1.63‒2.10; nonsevere hepatic disorder; ROR 4.27, 95% CI 3.68‒4.95; IC 1.83, 95% CI 1.62‒2.04). Tolvaptan was significantly associated with severe hepatic disorder and nonsevere hepatic disorder in both databases.

**Table 2:** Association between severe or nonsevere hepatic disorder and tolvaptan treatment.

	Cases	Noncases	ROR	95% CI	IC	95% CI
FAERS						
Severe hepatic disorder	265	1636	4.93	4.33‒5.61	2.11	1.92‒2.29
Nonsevere hepatic disorder	321	1580	6.78	6.01‒7.65	2.51	2.33‒2.68
JADER						
Severe hepatic disorder	167	952	4.21	3.57‒4.97	1.86	1.63‒2.10
Nonsevere hepatic disorder	219	900	4.27	3.68‒4.95	1.83	1.62‒2.04

FAERS; FDA Adverse Event Reporting System, JADER; Japanese Adverse Drug Event Report database, ROR; reporting odds ratio, CI; confidence interval, IC; information component.

### Multiple logistic regression analysis

Table 3 shows the results of multiple logistic regression analysis of severe or nonsevere hepatic disorder in patients treated with tolvaptan using variables of age and sex in FAERS and JADER. The nonsevere hepatic disorder was significantly associated with patients aged < 60 years in both databases (FAERS: adjusted ROR 2.90, 95% CI 2.26‒3.71, *p* < 0.0001; JADER: adjusted ROR 3.77, 95% CI 2.72‒5.23, *p* < 0.0001). In JADER, the nonsevere hepatic disorder was also significantly associated with the female sex (adjusted ROR 1.41, 95% CI 1.04‒1.92, *p* = 0.0283).

**Table 3:** Association between severe or nonsevere hepatic disorder and age or sex in patients treated with tolvaptan.

	Cases	Noncases	Adjusted ROR	95% CI	*p* value
FAERS					
Severe hepatic disorder					
Age (< 60 years)	107	644	1.04	0.80‒1.36	0.7547
Sex (female)	118	730	1.00	0.77‒1.29	0.9829
Nonsevere hepatic disorder					
Age (< 60 years)	196	555	2.90	2.26‒3.71	< 0.0001
Sex (female)	144	704	1.04	0.81‒1.33	0.7692
JADER					
Severe hepatic disorder					
Age (< 60 years)	41	189	1.30	0.89‒1.92	0.1786
Sex (female)	61	385	0.86	0.61‒1.20	0.3680
Nonsevere hepatic disorder					
Age (< 60 years)	89	141	3.77	2.72‒5.23	< 0.0001
Sex (female)	99	347	1.41	1.04‒1.92	0.0283

The adjusted ROR and 95% CI of severe or nonsevere hepatic disorder were determined based on age and sex.

FAERS; FDA Adverse Event Reporting System, JADER; Japanese Adverse Drug Event Report database, ROR; reporting odds ratio, CI; confidence interval.

### Time‑to‑onset analysis

**Fig. 2:**
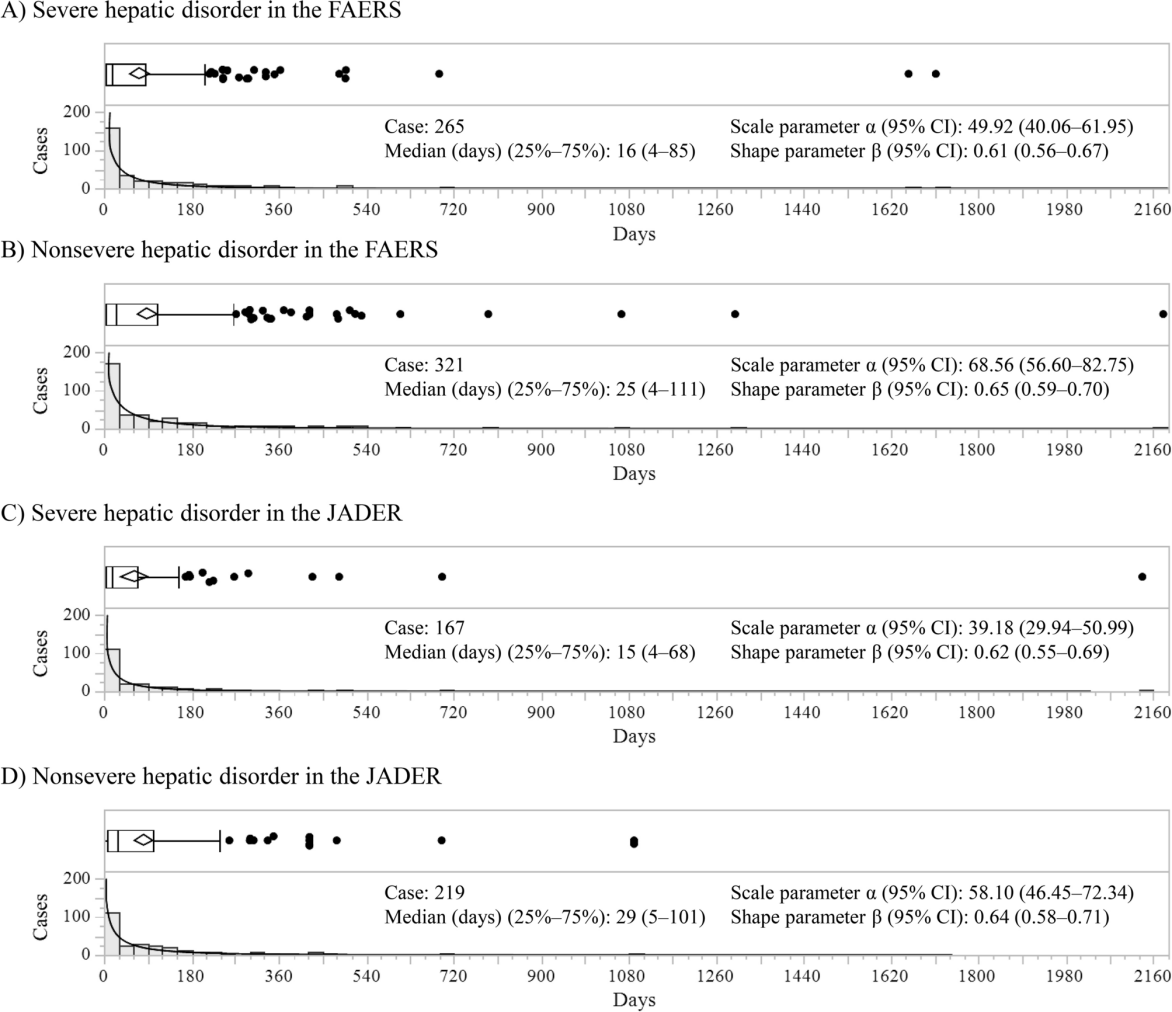
Histogram and Weibull shape parameters of hepatic disorder for severity in the FAERS and JADER. Upper panel shows the box plots, which represent the median (the vertical line within the box). The ends of the box represent the 1st and 3rd quartile, respectively. The confidence diamond contains the mean and the upper and lower 95% CIs of the mean. The whiskers extend to the outermost data point that falls within the distances representing 1.5-fold the interquartile range.

**Fig. 3:**
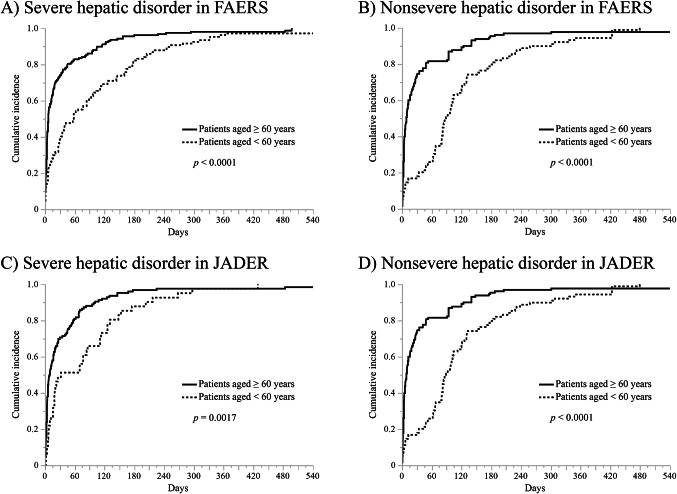
Kaplan–Meier curves for the incidence of hepatic disorder in patients aged < 60 years or ≥ 60 years based on severity of hepatic disorder in the FAERS and JADER. The x axis is limited to 540 days. Patients aged < 60 years had a significantly later onset of severe or nonsevere hepatic disorder than those aged ≥ 60 in the FAERS and JADER.

The results of the Weibull shape parameter test for severe or nonsevere hepatic disorder in the FAERS and JADER are shown in Fig. 2. In both databases, severe hepatic disorder and nonsevere hepatic disorder were considered to be the early failure type because the upper limit of the 95% CI of the β value is less than 1. Also, severe hepatic disorder and nonsevere hepatic disorder had the highest frequency of onset within 30 days [FAERS: severe hepatic disorder 59.6% (158/265 cases); nonsevere hepatic disorder 53.0% (170/321 cases), JADER: severe hepatic disorder 65.3% (109/167 cases); nonsevere hepatic disorder 50.7% (111/219 cases)]. Fig. 3. shows the Kaplan–Meier curves for age and severe or nonsevere hepatic disorder in the FAERS and JADER, respectively. In both databases, the median onset time of severe hepatic disorder and nonsevere hepatic disorder was significantly longer in patients aged < 60 years compared with that in patients aged ≥ 60 (FAERS: severe hepatic disorder 7 vs. 58 days, *p* < 0.0001; nonsevere hepatic disorder 8 vs. 52.5 days, *p* < 0.0001; JADER: severe hepatic disorder 9.5 vs. 32 days, *p* = 0.0017; nonsevere hepatic disorder 9 vs. 89 days, *p* < 0.0001).

## Discussion

In the present study, disproportionality analysis revealed that not only severe hepatic disorder, but also nonsevere hepatic disorder was related to tolvaptan. Our results suggest that tolvaptan is associated with hepatic disorder, regardless of severity. There have been reports of nonsevere hepatic disorder progressing to severe hepatic disorder^13,15^. Thus, it may be important to monitor both severe and nonsevere hepatic disorder in patients treated with tolvaptan.

Multiple logistic regression analysis in patients treated with tolvaptan revealed that nonsevere hepatic disorder was significantly associated with age < 60 years. Moreover, severe hepatic disorder was not significantly associated with age. Our results indicate that severe hepatic disorder occurs regardless of age, but nonsevere hepatic disorder tends to occur in nonelderly patients. In the FAERS and JADER, congenital cystic kidney disease accounted for the majority of all indications in individuals with nonsevere hepatic disorder. Congenital cystic kidney disease is prevalent in nonelderly patients^35^. Furthermore, high doses of tolvaptan are utilized in the treatment of congenital cystic kidney disease than in other indications such as heart failure, cirrhosis, and syndrome of inappropriate secretion of antidiuretic hormone^2,36^. Therefore, high doses of tolvaptan in nonelderly patients may have influenced the onset of nonsevere hepatic disorder. However, the incidence of congenital cystic kidney disease in all indications is low, and many reports are uncertain. Therefore, more research is needed to explain the relationship between nonsevere hepatic disorder and nonelderly patients.

The incidence of tolvaptan-induced hepatic disorder may be associated with female sex, but this remains unknown^15^. In the present study, sex was not related to severe hepatic disorder in the FAERS and JADER. Also, nonsevere hepatic disorder in the FAERS was not associated with sex. However, the female sex was associated with nonsevere hepatic disorder in JADER. The findings indicate that there may be an association between sex and nonsevere hepatic disorder in Japanese patients. Because JADER contains fewer spontaneous reports compared with FAERS, additional reports will be required to interpret the association between sex and nonsevere hepatic disorder.

The Weibull shape parameter test for severe and nonsevere hepatic disorder was considered to be the early failure type. This suggests that hepatic disorder caused by tolvaptan tends to occur early after starting treatment, regardless of severity. In previous reports, hepatic disorder in patients treated with tolvaptan appears to occur primarily after 2 months of starting treatment^10,14,37^. Meanwhile, our results indicate that the incidence of hepatic disorder within 1 month was high in FAERS and JADER, regardless of severity. Thus, our results are not consistent with previous reports. Because FAERS and JADER are databases based on spontaneous reports, our results may have been affected by various biases, such as the Weber effect, notoriety effect, and masking effect^38–40^. It is noteworthy; however, that we obtained similar results despite the different databases. To increase the reliability of this study, we analyzed different databases, FAERS and JADER, compared with previous studies^22–26^. Therefore, patients treated with tolvaptan should be closely monitored for the occurrence of hepatic disorder within 1 month of administration. The proportion of patients with elevated liver enzyme levels reached a plateau at approximately 350 days^10^. In addition, the onset of hepatic disorder has been reported to occur 3 to 18 months after the initiation of tolvaptan treatment^15^. These reports support our results, in which hepatic disorder occurred within 18 months in most patients. Recently, it was reported that the appropriate frequency of long-term hepatic monitoring was monthly during the first 18 months of tolvaptan exposure and every 3 months thereafter^10^. Considering our results, it appears reasonable to reduce the frequency of hepatic monitoring after 18 months.

The onset profiles of hepatic disorder indicated that nonelderly patients had a significantly later onset of severe or nonsevere hepatic disorder compared with elderly patients in FAERS and JADER. This result suggests that hepatic disorder occurs later in nonelderly patients compared with the elderly patients, regardless of the severity of hepatic disorder. We also examined trends in hepatic disorder outcomes, and the results indicated severe outcomes including “death,” “life-threatening,” “hospitalization,” and “not recovered” were included. In particular, severe hepatic disorder in elderly patients tended to be associated with “death.” Furthermore, even nonsevere hepatic disorder in nonelderly patients tended to result in “hospitalization” and “not recovered.” Previous studies found that patients with severe hepatic disorder may require hospitalization or even die^12,15,41^; however, the effects of age and severity of the hepatic disorder on outcomes have not been investigated due to the rarity of AEs. We found that even nonsevere hepatic disorder in nonelderly patients can cause severe outcomes, whereas severe hepatic disorder in elderly patients results in more severe outcomes. Liver function tests are recommended in patients treated with tolvaptan^10^, but the effects of age are not considered. Because hepatic disorder occurs later in nonelderly patients compared with elderly patients, liver function tests may need to be adjusted based on age to prevent fatal outcomes.

Some potential limitations of this study should be considered when interpreting the results. First, the FAERS and JADER are based on spontaneous AE reports, which may contribute to bias^38–40^. Also, it is impossible to catch all AEs that occur because spontaneous AE reports are voluntary. Furthermore, in addition to tolvaptan-induced hepatotoxicity, there may have been other AEs that led to liver injury but were not reported as liver related AEs. Second, we could not investigate the effect of concomitant drugs on the risk of tolvaptan-induced hepatotoxicity because tolvaptan is primarily metabolized by cytochrome P450 3A and is a substrate and inhibitor of P-glycoprotein, which could result in a variety of pharmacokinetic drug interactions^2^. Third, we were unable to assess safety information in patients aged < 20 years. The proportion of patients aged < 20 years was less than 1% in the FAERS and JADER, independent of severity. Finally, the relationship between indications for tolvaptan and liver injury was not assessed. The age at onset of disorder for which tolvaptan is recommended varies per disease^35,42–44^. Therefore, the discrepancy in indicators may have influenced the timing of onset. However, there were numerous reports with unclear indications in the FAERS and JADER. Also, the grounds for using tolvaptan included symptoms as well as indications. Thus, we were unable to evaluate the association between tolvaptan and liver injury based on indications. Nonetheless, this large-scale pharmacovigilance analysis provides useful information for hepatic monitoring in patients treated with tolvaptan.

In this study, we focused on hepatic disorder caused by tolvaptan. Tolvaptan administration was related to severe as well as nonsevere hepatic disorder. Also, hepatic disorder occurred significantly later in nonelderly patients compared with elderly patients, regardless of the severity of hepatic disorder. In addition, severe and nonsevere hepatic disorder can result in severe outcomes. Therefore, patients treated with tolvaptan should be monitored for hepatic function based on age to prevent fatal outcomes.

## Supplementary Information

Supplementary Information.

## Data Availability

The FAERS and JADER databases analyzed in this study are publicly available. Both databases can be downloaded from their websites (FAERS: https://www.fda.gov/, JADER: https://www.pmda.go.jp/index.html).
