# Supplementary material for: Evaluation of tolvaptan-associated hepatic disorder using different national pharmacovigilance databases
Source: Sci Rep. 2024 Oct 29;14:25943. doi: 10.1038/s41598-024-77052-y (PMC11522566; doi:10.1038/s41598-024-77052-y)
Supplement: Supplementary file 1 — Supplementary Information. [file 41598_2024_77052_MOESM1_ESM.pdf]

Supplementary Information

Evaluation of tolvaptan-associated hepatic disorder using different national pharmacovigilance databases

Journal: Scientific Reports

Authors: Takaya Uno, Kouichi Hosomi, Satoshi Yokoyama

Corresponding author: Takaya Uno (uno@phar.kindai.ac.jp)

Supplementary Table S1. Indications for severe hepatic disorders related to tolvaptan

FAERS	Value (%)	JADER	Value (%)
Congenital cystic kidney disease	43 (16.2)	Fluid retention	35 (20.5)
Fluid retention	39 (14.7)	Edema due to hepatic disease	25 (14.6)
Cardiac failure	17 (6.4)	Cardiac failure	20 (11.7)
Cardiac failure congestive	13 (4.9)	Congenital cystic kidney disease	18 (10.5)
Edema due to hepatic disease	10 (3.8)	Hepatic cirrhosis	13 (7.6)
Ascites	9 (3.4)	Ascites	11 (6.4)
Hepatic cirrhosis	9 (3.4)	Cardiac failure congestive	10 (5.8)
Cardiac failure acute	4 (1.5)	Cardiac failure chronic	8 (4.7)
Hyponatremia	4 (1.5)	Edema due to cardiac disease	3 (1.8)
Cardiac failure chronic	3 (1.1)	Cardiac failure acute	2 (1.2)
Edema due to cardiac disease	3 (1.1)	Edema	2 (1.2)
Inappropriate antidiuretic hormone secretion	2 (0.8)	Fluid imbalance	2 (1.2)
Pleural effusion	2 (0.8)	Pleural effusion	2 (1.2)
Cirrhosis alcoholic	1 (0.4)	Cirrhosis alcoholic	1 (0.6)
Congenital renal disorder	1 (0.4)	Hepatic failure	1 (0.6)
Diabetic nephropathy	1 (0.4)	Hepatic hydrothorax	1 (0.6)
Edema peripheral	1 (0.4)	Hyponatremia	1 (0.6)
Gastric cancer	1 (0.4)	Polyuria	1 (0.6)
Hypoosmolar state	1 (0.4)	Portopulmonary hypertension	1 (0.6)
Ill-defined disorder	1 (0.4)	Primary biliary cholangitis	1 (0.6)
Left ventricular failure	1 (0.4)	Pulmonary edema	1 (0.6)
Polyuria	1 (0.4)	Unknown	12 (7.0)
Renal failure	1 (0.4)		
Right ventricular failure	1 (0.4)		
Unknown	96 (36.2)		

Because patients reported two indications, the total number of indications does not always correspond to the total number of patients.

FAERS; FDA Adverse Event Reporting System, JADER; Japanese Adverse Drug Event Report database

Supplementary Table S2. Indications for nonsevere hepatic disorders related to tolvaptan

FAERS	Value (%)	JADER	Value (%)
Congenital cystic kidney disease	65 (20.2)	Congenital cystic kidney disease	67 (30.3)
Cardiac failure	20 (6.2)	Cardiac failure	38 (17.2)
Fluid retention	14 (4.3)	Cardiac failure congestive	18 (8.1)
Hyponatremia	8 (2.5)	Fluid retention	16 (7.2)
Edema due to cardiac disease	6 (1.9)	Cardiac failure chronic	11 (5.0)
Cardiac failure congestive	4 (1.2)	Edema due to hepatic disease	7 (3.2)
Hepatic cirrhosis	4 (1.2)	Edema due to cardiac disease	6 (2.7)
Cardiac failure chronic	3 (0.9)	Ascites	3 (1.4)
Polyuria	3 (0.9)	Cirrhosis alcoholic	2 (0.9)
Congestive cardiomyopathy	2 (0.6)	Edema peripheral	2 (0.9)
Edema due to hepatic disease	2 (0.6)	Hepatic cirrhosis	2 (0.9)
Bile duct cancer	1 (0.3)	Hyponatremia	2 (0.9)
Cardiac dysfunction	1 (0.3)	Polyuria	2 (0.9)
Cardiomyopathy	1 (0.3)	Right ventricular failure	2 (0.9)
Chronic kidney disease	1 (0.3)	Cardiac failure acute	1 (0.5)
Cirrhosis alcoholic	1 (0.3)	Edema due to renal disease	1 (0.5)
Edema peripheral	1 (0.3)	Generalized edema	1 (0.5)
Generalized edema	1 (0.3)	Inappropriate antidiuretic hormone secretion	1 (0.5)
Hypervolemia	1 (0.3)	Infection	1 (0.5)
Inappropriate antidiuretic hormone secretion	1 (0.3)	Renal failure	1 (0.5)
Nephrotic syndrome	1 (0.3)	Renal impairment	1 (0.5)
Polycystic kidney, autosomal dominant	1 (0.3)	Unknown	36 (16.3)
Rapid correction of hyponatremia	1 (0.3)		
Right ventricular failure	1 (0.3)		
Unknown	178 (55.3)		

Because patients reported two indications, the total number of indications does not always correspond to the total number of patients.

FAERS; FDA Adverse Event Reporting System, JADER; Japanese Adverse Drug Event Report database
